# Electron tomography and immunonanogold electron microscopy for investigating intracellular trafficking and secretion in human eosinophils

**DOI:** 10.1111/j.1582-4934.2008.00346.x

**Published:** 2008-04-10

**Authors:** Rossana C N Melo, Ann M Dvorak, Peter F Weller

**Affiliations:** a Laboratory of Cellular Biology, Department of Biology Federal University of Juiz de Fora, UFJFJuiz de Fora, Minas Gerais, Brazil; b Department of Pathology Beth Israel Deaconess Medical Center, Harvard Medical SchoolBoston, MA, USA; c Department of Medicine, Beth Israel Deaconess Medical Center, Harvard Medical SchoolBoston, MA, USA

**Keywords:** electron tomography, immunonanogold electron microscopy, cell secretion, vesicular transport, eosinophils, inflammation, eosinophil sombrero vesicles (EoSVs)

## Abstract

Electron tomography (ET) has increasingly been used to understand the complexity of membrane systems and protein-trafficking events. By ET and immunonanogold electron microscopy, we recently defined a route for vesicular transport and release of granule-stored products from within activated human eosinophils, cells specialized in the secretion of numerous cytokines and other proteins during inflammatory responses. Here, we highlight these techniques as important tools to unveil a distinct eosinophil vesicular system and secretory pathway.

The classical picture of the cell secretory pathway includes protein synthesis within the endoplasmic reticulum, transport of cargo inwards towards the Golgi apparatus and then through the Golgi and *trans*-Golgi network *en routeto* the plasma membrane, all carried by the transport vesicles [1]. Human eosinophils, leucocytes of the innate immune system, with functions in allergic, inflammatory and immunoregulatory responses [2], additionally show a novel secretory pathway organization (reviewed in Reference [3]).

Recent studies based on fully automated electron tomography (ET) and refined immunonanogold electron microscopy (EM) revealed that, during eosinophil secretion, a distinct population of large, tubular transport vesicles, termed eosinophil sombrero vesicles (EoSVs) (Fig. 1), bud from the cytoplasmic secretory granules (also referred to as specific granules), and in conjunction with small, round vesicles, transport granule-stored products to the plasma membrane for extracellular release [4]. This vesicle-mediated process of cell secretion (piecemeal degranulation) [5], also frequently identified in other cells [6, 7], was until recently believed to be accomplished only by small, round vesicles. However, recent data have provided conclusive evidence for both the active formation of large vesiculotubular carriers (EoSVs) in response to cell activation (Figs. 1 and 2A) and their participation in granule-to-plasma membrane trafficking [4]. Combining pre-embedding immunonanogold EM for precise epitope preservation, highly specific monoclonal antibodies and subcellular localization associated with very small gold particles (1.4 nm) as a probe, EoSVs were positively immunola-belled for typical granule products such as major basic protein (MBP) (Fig. 2B and C) and interleukin 4 (IL-4) [4, 8]. MBP is one of the most abundant cationic proteins stored within and recognized as a marker of the eosinophil specific granules, while IL-4, also stored in the specific granules, is a hallmark eosinophil cytokine, typical of allergic and anti-helminthic parasite immune responses [9].

**Fig. 1:**
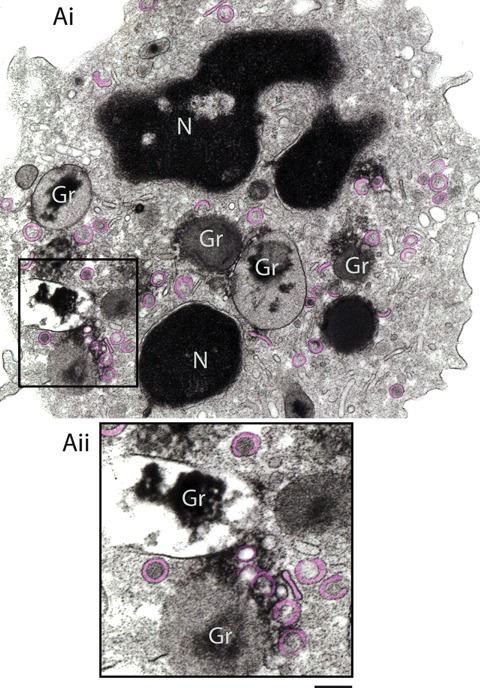
Ultrastructural image of a human activated eosinophil. (**Ai**) Eosinophil sombrero vesicles (EoSVs) (lumens highlighted in pink) with typical morphology are observed in the cytoplasm by transmission electron microscopy. These vesicles show a ‘Mexican hat’ (sombrero) appearance or a ‘C’-shaped morphology in conventional cross-thin sections (∼80 nm of thickness) of the eosinophils. Secretory granules (Gr), seen in progressive stages of emptying, indicate occurrence of piecemeal degranulation. (**Aii**) is the boxed area of (**Ai**) and shows in higher magnification several EoSVs profiles in close apposition to a mobilized granule. Eosinophils, isolated from the blood by negative selection [8], were stimulated with recombinant stem cell factor and processed as described in Reference [4]. N, nucleus. Bars: 630 nm (**Ai**), 300 nm (**Aii**).

**Fig. 2:**
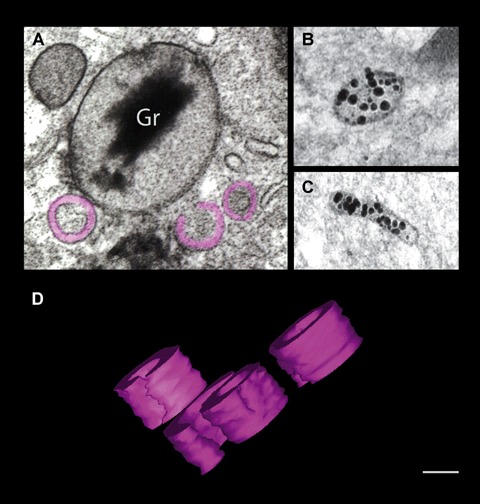
Eosinophil sombrero vesicles (EoSVs) are open, tubular-shaped carriers actively involved in the eosinophil secretory pathway. (**A**) An image from conventional transmission electron microscopy (TEM) shows EoSVs (lumens highlighted in pink) around a secretory granule (Gr) with a disarranged core. In (**B**) and (**C**) EoSVs within activated eosinophils are immunolabelled for major basic protein (MBP). In (**D**), a three-dimensional (3D) model generated from 4-nm thick serial slices by electron tomography shows EoSVs as curved, tubular and open structures surrounding a cytoplas-mic centre. The cells were stimulated with stem cell factor (**A**) or eotaxin (**B–D**) and processed for conventional TEM or immunonanogold EM as before [4]. Tilt series were acquired, fully automatically at 200 kV, on a FEI Tecnai Sphera microscope (FEI's Nanoport-Eidhoven, The Netherlands). Tomograms were generated using Xplore 3D software (FEI) [4]. Modelling was carried out using IMOD software (The Boulder Laboratory for 3-D Electron Microscopy of Cells, University of Colorado) [15]. Bars: 250 nm (**A**), 180 nm (**B** and **C**), 150 nm (**D**).

Studies performed with ET and computer-based modelling, powerful approaches for understanding cellular architecture [10], revealed the three-dimensional (3D) structure of EoSVs (Fig. 2D) [4]. They are folded, flattened tubular carriers, larger (150–300 nm in diameter) and more pleiomorphic than the conventional small (∼50 nm in diameter), spherical vesicles. EoSVs present substantial membrane surfaces (Fig. 2D) and represent a dynamic system with a remarkable ability to change their shape and to interact with the secretory granules (Figs. 1 and 2A) [3, 4]. The curved morphology of EoSVs provides a higher surface-to-volume ratio system, likely important for the specific transport of the membrane-bound proteins. In fact, further studies by our group confirmed that these vesicles transport IL-4 through a membrane-bound receptor-mediated mechanism [11]. This, importantly, might underlie the remarkable ability of the eosinophils to participate in distinct immune responses [12].

Studies focussed on the mechanisms of eosinophil secretion are critical not only to understand the normal leucocyte function, but also to understand the pathological basis of allergic and inflammatory diseases. Moreover, the identification of EoSVs as important secretory vesicles within the eosinophils is defining a broader role for large vesiculotubular carriers in intracellular trafficking and secretion of proteins, as described in different cell secretory pathways [3, 13, 14].
